# Editorial: Digital media use and mental health

**DOI:** 10.3389/fpsyt.2023.1253846

**Published:** 2023-07-26

**Authors:** Rosa S. Wong, Keith T. S. Tung, Frederick K. W. Ho, Kenneth K. C. Man

**Affiliations:** ^1^Department of Paediatrics and Adolescent Medicine, The University of Hong Kong, Pok Fu Lam, Hong Kong SAR, China; ^2^Institute of Health and Wellbeing, University of Glasgow, Glasgow, United Kingdom; ^3^Research Department of Practice and Policy, UCL School of Pharmacy, London, United Kingdom; ^4^Department of Pharmacology and Pharmacy, Centre for Safe Medication Practice and Research, The University of Hong Kong, Pok Fu Lam, Hong Kong SAR, China

**Keywords:** digital media, mental health, social media, video gaming, screen, person-specific analyses

Digital media has become a crucial component of modern life. People of all ages and professions, not just the youth, are utilizing digital media for communication and entertainment. The impact of digital media use on mental health can be positive or negative, partly depending on an individual's susceptibility to the content (1, 2). Variations in susceptibility can make it challenging to implement effective prevention and intervention strategies (3). It is thus important to understand which, when, how, and why individuals may be influenced by certain types of media.

As researchers investigating the interplay of public health and media, our primary focus is on examining the ways in which different subsets of the population are affected positively and negatively in terms of mental health by media exposure, given that the link between digital technology use and psychological wellbeing is not always negative (4). Indeed, there is research indicating that spending more time on social media does not necessarily lead to increased mental health issues across development stages when analyzed at the individual level (5). Therefore, in February 2022, we issued a call for the Research Topic “*Digital Media Use and Mental Health*” with the goal of gathering up-to-date evidence and encouraging further research into understanding the various factors, including the context and content of social media use, that may contribute to the rise of mental health issues during different developmental periods.

This Research Topic brings together seven papers by 49 authors from both western and non-western regions, all of which were original research studies. Taken together, this body of work reveals the significance of dispositional (person-specific) factors in determining the mental health consequences of digital media use. Although all the papers focus on adolescents and young adults, each group examined has unique characteristics and online experiences that account for differences in their emotional responses to various online activities. Among papers that focus on vulnerable subgroups, there is one that details how autistic youth use social media (Leung et al.), another that explores the online experiences of lesbian, gay, bisexual, transgender, and queer (LGBTQ) youth (Escobar-Viera et al.), and two that center on cyberbullying victims (Chu et al.; Zhang et al.). Their results show that despite a clear connection between cyberbullying victimization and depression (Chu et al.; Zhang et al.), it is possible for underrepresented/disadvantaged groups to reap benefits from utilizing social media if the online community is supportive and provides a safe environment (Escobar-Viera et al.; Leung et al.). For other individuals, pre-existing vulnerabilities such as limited offline social activities (Lyyra et al.), disturbed sleep patterns (Li et al.), and sensitivity to negative feedback (Piksa et al.) may also make them more prone to the adverse effects of digital media use.

Although there is a proliferating literature on the link between screen time and mental health, the quality of screen use in terms of context and content also matters. A strength of this Research Topic is that it consolidates viewpoints from a variety of online users, which aids in exploring the circumstances, reasons, and groups for whom the utilization of digital media can have positive or negative effects on mental health. This is an important starting point in comprehending the impact of digital media use on mental health. However, since most research studies provide aggregated data (2), future research should delve into person-specific causes and outcomes of digital media use to establish a more comprehensive framework for understanding its mental health effects.

## Author contributions

RW, KT, FH, and KM equally contributed to the editorial process. RW organized the group. All authors contributed to requesting submissions, reviewing, and editing manuscripts.
